# Functional interplay between E2F7 and ribosomal rRNA gene transcription regulates protein synthesis

**DOI:** 10.1038/s41419-018-0529-6

**Published:** 2018-05-14

**Authors:** Amanda S Coutts, Shonagh Munro, Nicholas B La Thangue

**Affiliations:** 1 Laboratory of Cancer Biology, Department of Oncology, Medical Sciences Division, University of Oxford, Old Road Campus Research Building, Old Road Campus, Off Roosevelt Drive, Oxford, OX3 7DQ UK; 2 Present Address: College of Science and Technology, Nottingham Trent University, Clifton Lane, Nottingham, NG11 8NS UK

## Abstract

A prerequisite for protein synthesis is the transcription of ribosomal rRNA genes by RNA polymerase I (Pol I), which controls ribosome biogenesis. UBF (upstream binding factor) is one of the main Pol I transcription factors located in the nucleolus that activates rRNA gene transcription. E2F7 is an atypical E2F family member that acts as a transcriptional repressor of E2F target genes, and thereby contributes to cell cycle arrest. Here, we describe an unexpected role for E2F7 in regulating rRNA gene transcription. We have found that E2F7 localises to the perinucleolar region, and further that E2F7 is able to exert repressive effects on Pol I transcription. At the mechanistic level, this is achieved in part by E2F7 hindering UBF recruitment to the rRNA gene promoter region, and thereby reducing rRNA gene transcription, which in turn compromises global protein synthesis. Our results expand the target gene repertoire influenced by E2F7 to include Pol I-regulated genes, and more generally suggest a mechanism mediated by effects on Pol I transcription where E2F7 links cell cycle arrest with protein synthesis.

## Introduction

The rate of protein synthesis is directly proportional to cell growth and proliferation. This is, in turn, intimately linked to ribosome biogenesis, which is controlled at the transcription level by Pol I^1^. The Pol I transcription machinery integrates information from cellular signalling cascades to regulate ribosome production and this guides cell growth and proliferation^1^. Ribosome biogenesis occurs in the nucleolus, and transcription of rRNA genes by Pol I is a major point of control. Pol I accounts for up to 60% of transcriptional activity in the cell, and rRNA contributes for up to 80% of the total RNA^2^. Interestingly, ribosome biosynthesis consumes about 80% of a cell’s energy and nearly all metabolic and signalling pathways lead to or from the nucleolus^3^.

The eukaryotic ribosome has a core complex of about 80 proteins and four rRNAs. The mature 80S ribosome is comprised of a large (60S) and a small (40S) subunit; the large subunit contains the 28S, 5.8S and 5S rRNAs, while the small subunit contains the 18S rRNA^4^. In humans, the 28S, 5.8S and 5S rRNA molecules are encoded within tandemly repeated 47 kb nucleolar-organising regions (NORs), which reside on the five acrocentric chromosomes, while the 5S rRNA is encoded by a tandemly repeated cluster on chromosome 1^5,6^. NORs are essential for nucleolar structure where they localise to form the nucleolus^7^.

During proliferation, cellular stress and differentiation, cells downregulate the synthesis of rRNA and ribosome biogenesis, thus designating the nucleolus as a central hub that coordinates cellular growth^1,8^. Ribosome biogenesis is tightly regulated by key proteins involved in cell growth and proliferation. For example, tumour suppressor proteins, such as p53, influence ribosome biogenesis in a negative fashion through interfering with the Pol I transcription factors, UBF and SL-1^9^. Conversely, oncoproteins, such as MYC, locate to the rRNA promoter to enhance Pol I activity^10^. In this way, ribosome biogenesis is intimately connected with cell growth and proliferation, and is affected by oncogenic events that occur in tumour suppressor and growth-promoting pathways. Importantly, the nucleolus and Pol I activity are increasingly viewed as attractive therapeutic targets, as proliferative cells are dependent on ribosome biogenesis for growth^11,12^.

E2F is a generic term for a family of master regulators that co-ordinate transcription with cell cycle progression^13^. E2F is a key target for the retinoblastoma tumour suppressor pRb, and deregulation of the pathway is of primary importance in proliferative disease like cancer, where aberrant pRb activity occurs through a variety of oncogenic mechanisms^13^. The E2F family has eight distinct members, with E2F7 regarded as atypical because it is endowed with pRb-independent repressive activity, which it exerts on E2F target genes leading to cell cycle arrest^14–17^. In addition, during the DNA damage response, E2F7 activity is upregulated where it impacts on cell cycle progression and DNA repair^18,19^.

Here, we describe an unexpected and surprising role for E2F7 in regulating ribosomal gene transcription. Thus, we have found that E2F7 localises to the nucleolar cap region, a major site of rRNA synthesis, which is dependent on its DNA-binding activity. E2F7 located to the Pol I promoter, and silencing E2F7 led to enhanced recruitment of UBF and thereafter increased Pol I activity. Accordingly, E2F7 affects global cellular protein synthesis in a negative fashion. Our results provide the first evidence that links E2F7 activity with ribosomal biogenesis, and thereby provide a mechanism for integrating cell cycle progression with cell growth and protein synthesis.

## Results

### E2F7 localises to the nucleolus

**Fig. 1:**
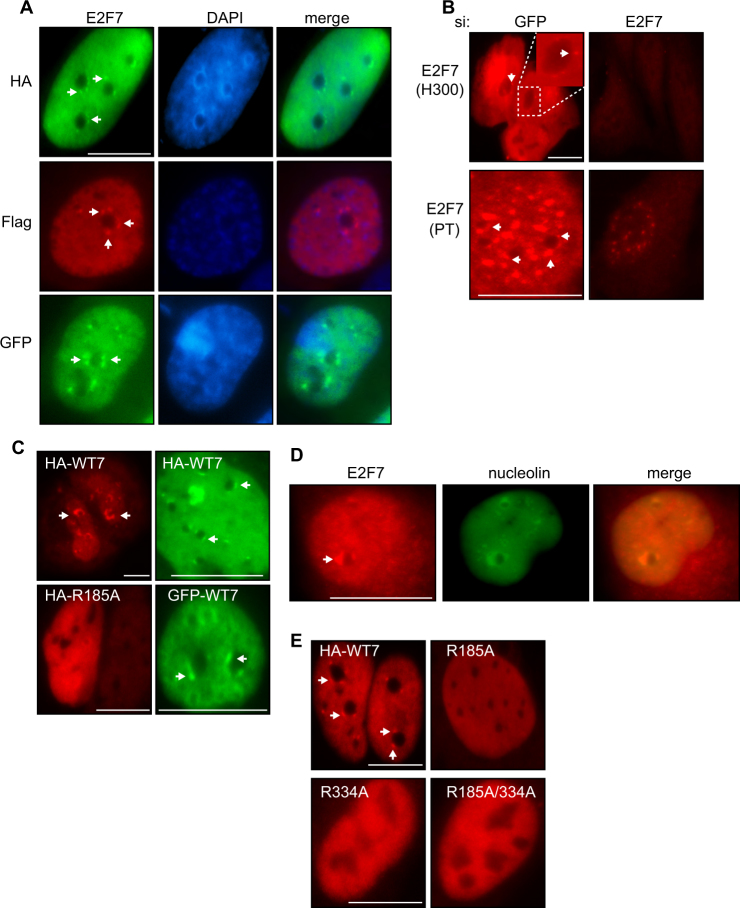
E2F7 localises to the nucleolus. **a** U2OS cells expressing HA, Flag or GFP-tagged E2F7. HA-E2F7 and Flag-E2F7 were detected with HA and Flag antibodies, respectively. DAPI staining was used to visualise nuclei. **b** Endogenous E2F7 was detected in asynchronously growing U2OS cells using rabbit anti-E2F7 antibody H300 (Santa Cruz) and rabbit anti-E2F7 antibody PT (Proteintech). **c** U2OS cells expressing HA wild-type E2F7 (HA-WT7), GFP-E2F7 or DNA-binding domain mutant R185A were treated with 1 nM actinomycin D overnight. HA-E2F7 was detected with anti-HA antibody. **d** Endogenous E2F7 (rabbit anti-E2F7 PT) and nucleolin were detected in U2OS cells treated with 1 nM actinomycin D. **e** U2OS cells were transfected HA-E2F7 constructs containing wild-type E2F7 (HA-WT7) or the DNA-binding domain mutants R185A, R334A and the double-mutant R185A/R334A. Cells were treated with 1 nM actinomycin D overnight before fixation and processing for immunofluorescence. Ectopic E2F7 was detected with anti-HA antibody. Scale bars = 10 μm. Arrows indicate E2F7 perinucleolar localisation

In examining the intracellular location of E2F7, we observed that in addition to its expected nuclear localisation, E2F7 was present at nucleolar cap structures which we observed with both Flag and HA-tagged E2F7, as well as GFP-tagged E2F7 compared to GFP alone (Fig. 1a and SI Fig. 1A). Importantly, we examined the localisation of endogenous E2F7 using two different E2F7 antibodies. Similar to what was observed upon the expression of ectopic E2F7, endogenous E2F7 was present throughout the nucleus (Fig. 1b). Importantly, E2F7 could also be seen to localise to the perinucleolar region (Fig. 1b). It is known that cellular stress, such as DNA damage, and direct inhibition of Pol I, result in nucleolar segregation and the increased formation of nucleolar caps around the nucleolar remnant^8^. We therefore used low-dose actinomycin D treatment to inhibit Pol I activity^20^. Under these conditions, we observed enhanced localisation of E2F7 to nucleolar caps (Fig. 1c and SI Fig. 1A, B) and endogenous E2F7 colocalised in the perinucleolar region with the nucleolar protein nucleolin (Fig. 1d).

E2F7 contains a bi-partite DNA-binding domain, and its integrity is required for transcriptional repression^15,16^. Of note, we found that DNA-binding domain (DBD) point mutants, unable to act as transcriptional repressors (SI Fig. 1C), were not able to localise at the nucleolus, even in the presence of actinomycin D (Fig. 1c, e). The ability of E2F7 to act at the level of DNA binding is thus necessary for its nucleolar localisation.

### E2F7 inhibits Pol I activity

**Fig. 2:**
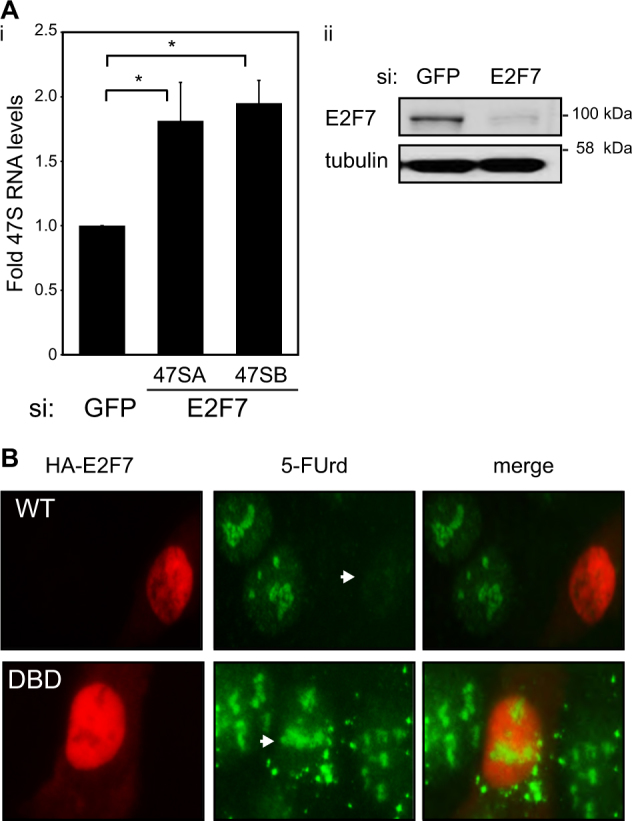
E2F7 inhibits Pol I activity. **a i** U2OS cells were treated with control (GFP) or E2F7 siRNA for 72 h before RNA isolation and RT-qPCR. 47S transcript levels were quantified using two different primer sets (A and B) and results are expressed as fold over control after normalising for GAPDH levels. Graph represents mean ± s.e.m., *n* = 3 independent experiments, *p < 0.05, Student’s *t* test. **ii** Blots represent the level of E2F7 and tubulin. **b** Nascent nucleolar RNA synthesis was detected after growing asynchronous U2OS cells transfected with HA wild-type E2F7 (WT) or the DNA-binding mutant R185A (DBD) in the presence of 2 mM 5-FUrd for 30 min before fixation and processing for immunofluorescence. RNA incorporated 5-FUrd was detected using anti-BrdU antibody BU-33 and E2F7 with anti-HA. Arrows highlight regions where there is a lack of FUrd incorporation (WT) or regions where FUrd incorporation is unaffected (DBD)

Since the nucleolar location of E2F7 is dependent upon its DNA-binding domain, we reasoned that E2F7 could impact on rRNA gene transcription. We therefore examined the effect on Pol I activity by silencing endogenous E2F7 and measuring 47S pre-rRNA transcript levels. The level of pre-rRNA was significantly increased after E2F7 depletion (nearly twofold, Fig. 2a). This was specific to ongoing Pol I activity as the presence of actinomycin D inhibited rRNA gene transcription (SI Fig. 2A). This suggested that E2F7 has a negative impact on Pol I activity. In support of this idea, we used 5-fluorouridine (FUrd) incorporation in situ to measure nascent RNA synthesis^20^, where we observed that in cells expressing ectopic E2F7 there was a marked loss of FUrd incorporation (Fig. 2b). In contrast, expression of a DBD mutant derivative E2F7 had little effect on nucleolar FUrd incorporation (Fig. 2b), confirming that the ability of E2F7 to inhibit Pol I activity and prevent rRNA synthesis required an intact DNA-binding domain.

**Fig. 3:**
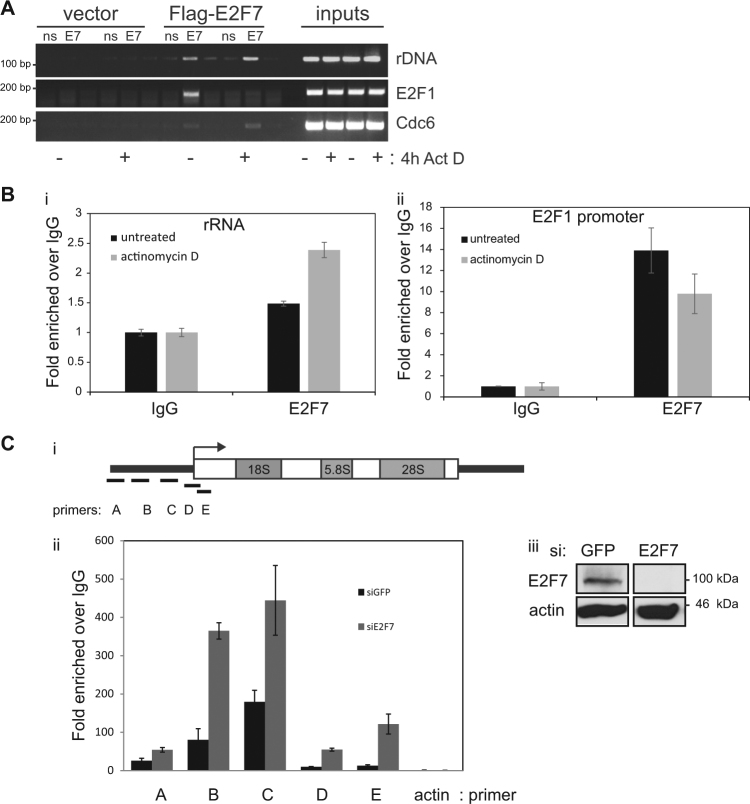
E2F7 localises to rRNA promoter. **a** U2OS cells were transfected with Flag-E2F7 or vector control. ChIP was performed cells with (+) and without (−) actinomycin D (ActD) treatment (20 nM, 4 h). PCR was performed using primers that amplify rDNA promoter −144 to +20. **b** An endogenous ChIP was performed in U2OS cells with (+) and without (−) actinomycin D (ActD) treatment (20 nM, 4 h). PCR was performed using primers that amplify rDNA promoter (**i**) −144 to +20, and the E2F1 promoter (**ii**). **c** ChIP was performed in U2OS cells treated for 72 h with either GFP siRNA or E2F7 siRNA using rabbit anti-UBF or rabbit IgG. **i** Schematic represents the Pol I promoter and the approximate positions of the primer sets used are shown. **ii** Graph represents range ±SD. qPCR was performed on ChIP chromatin using actin as a control gene. Results are expressed as fold over IgG (rabbit non-specific IgG) after normalising to input levels. *n* = 3 independent experiments. **iii** Blots on extracts demonstrating E2F7 depletion. E2F7 was detected using rabbit anti-E2F7 antibody and actin was used as a loading control

Next, we explored the possibility that E2F7 is present in the chromatin environment of the Pol I promoter. By chromatin immunoprecipitation (ChIP) we detected E2F7 at the Pol I promoter (Fig. 3a, b). The levels were similar to that of E2F7 at the E2F1 promoter under normal growth conditions, and markedly higher when compared to the E2F target gene CDC6 (Fig. 3a). Interestingly, the presence of E2F7 at the E2F1 promoter was significantly decreased when Pol I activity was prevented with actinomycin D, while its presence at the rRNA gene promoter remained unaffected (Fig. 3a, b). Since transcription factor UBF is essential for transcription by RNA Pol I^21,22^, we examined any effect of E2F7 on UBF recruitment to the Pol I promoter, and observed a significant enhancement in the recruitment of UBF to the rRNA gene promoter region after E2F7 depletion (Fig. 3c). Together, these results suggest that E2F7 acts negatively on Pol I rRNA gene transcription and that the integrity of its DNA-binding domain is required for this to occur.

### E2F7 inhibits total protein synthesis

**Fig. 4:**
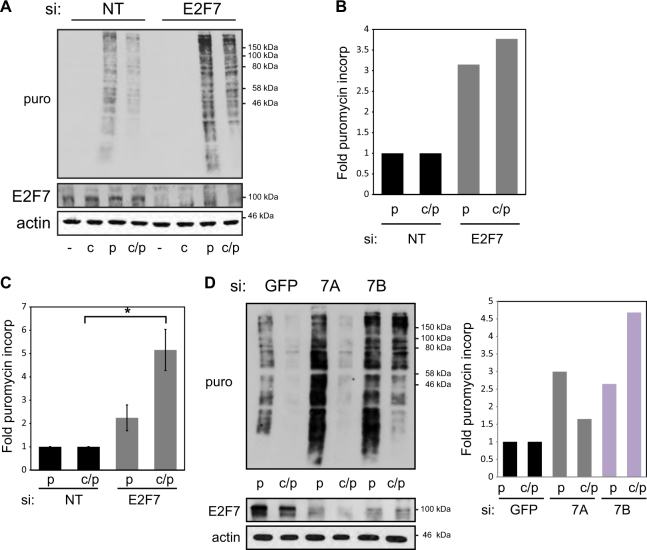
E2F7 influences protein synthesis. **a** U2OS cells were treated with control (NT) or E2F7 siRNA for 72 h before performing puromycin incorporation assays. Steady-state (puromycin = p) and nascent (puromycin/cycloheximide = c/p) protein levels were detected using anti-puromycin antibody. **b** Graph represents quantification of the level of puromycin incorporated in **a** after normalising for actin. **c** U2OS cells were treated as in **a**. Graph represents mean ± s.e.m., *n* = 3 independent experiments. *p* < 0.05, Student’s *t* test. **d** U2OS cells were treated with control (GFP) or E2F7 (A and B) siRNA for 72 h before performing puromycin incorporation assays. P puromycin, c/p cyclohexamide/puromycin. Graph represents the fold puromycin incorporation after normalising to actin. *n* = 3 independent experiments

RNA Pol I activity results in enhanced ribosome production leading to increased protein synthesis^23^. To explore what effect E2F7 has on total protein synthesis we performed puromycin incorporation assays to determine both steady-state and nascent protein production^24^. We found that E2F7 depletion resulted in a significant increase in the level of protein synthesis (Fig. 4a–c and SI Fig. 2B). To confirm the specificity of this effect we depleted E2F7 with two different siRNAs, both of which resulted in E2F7 depletion and enhanced protein synthesis (Fig. 4d and SI Fig. 2C). The ability of E2F7 to influence protein synthesis appeared not to be cell-type-specific as similar results were observed in both U2OS (osteosarcoma) and MCF7 (breast cancer) human cancer cell lines (Fig. 4a–d and SI Fig. 2B, C). Together, these results suggest a mechanism whereby E2F7 through its ability to downregulate Pol I transcription is able to affect global protein synthesis.

**Fig. 5:**
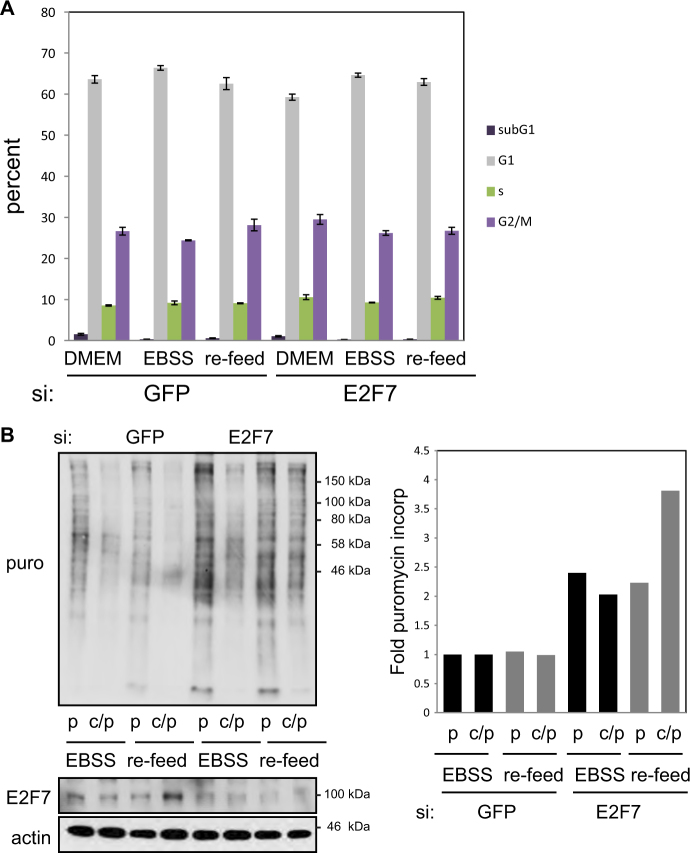
E2F7 effects on protein synthesis can be dissociated from cell cycle. **a** U2OS cells treated with either control (GFP) or E2F7 siRNA for 72 h. Cells were grown under starvation conditions for 4 h before being re-fed with full growth medium, DMEM (re-feed) for 1 h before being harvested for FACS analysis. Cells were set up in triplicate. **b** Puromycin incorporation assays were carried out on U2OS cells grown under starvation conditions for 4 h before being re-fed with full growth medium, DMEM (re-feed) for 1 h. Graph represents fold puromycin incorporation after normalising to actin levels (GFP EBSS control set as 1). *n* = 2 independent experiments

E2F7 influences cell cycle progression and is itself regulated in a cell cycle-dependent fashion^15–17,25^. Despite this, in asynchronously growing cells under conditions where no cell cycle perturbation upon E2F7 depletion was apparent (Fig. 5a), increased protein synthesis was observed (Fig. 4a–d), suggesting that the effect on protein synthesis was not influenced by significant alterations in cell cycle progression. Moreover, since cell starvation is a key regulator of protein synthesis^26^, we also examined if during short-term starvation E2F7 had any effect on protein synthesis. During a 4 h starvation (EBSS) with or without a 1 h re-feed, depleting E2F7 had no obvious effects on the cell cycle profile (Fig. 5a). Yet, under these conditions, E2F7 depletion resulted in a marked increase in protein synthesis (Fig. 5b), demonstrating the effect of E2F7 on Pol I activity, and thus protein synthesis, could be uncoupled from cell cycle progression.

### E2F7 causes nucleolar segregation

**Fig. 6:**
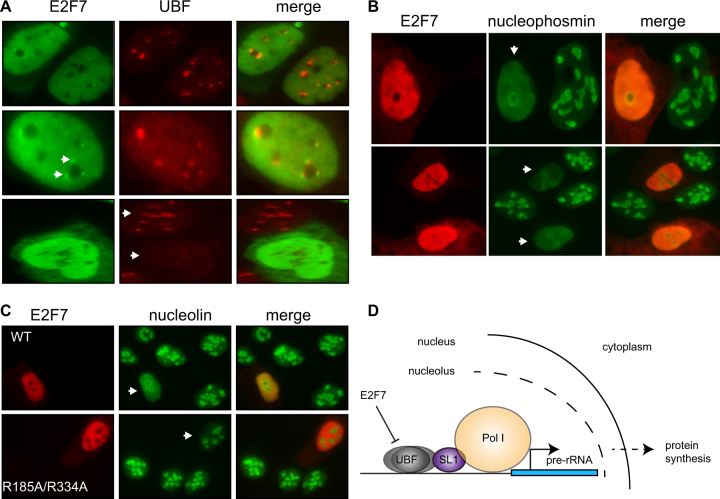
E2F7 results in nucleolar segregation. **a** U2OS cells expressing HA wild-type E2F7 (E2F7). E2F7 was detected using anti-HA antibody and endogenous UBF was detected with anti-UBF antibody. **b** U2OS cells expressing HA wild-type E2F7 (E2F7). Nucleophosmin was detected with anti-nucleophosmin antibody. **c** U2OS cells expressing HA wild-type E2F7 (E2F7) or double DNA-binding mutant R185A/R334A. Nucleolin was detected with anti-nucleolin antibody. **d** Diagram depicting a simplified model of UBF influence on Pol I activity resulting in increased pre-rRNA gene expression leading to enhanced protein synthesis. Elevated levels of perinucleolar E2F7 results in E2F7 binding to the rRNA promoter resulting in decreased pre-rRNA expression leading to decreased protein synthesis

We noted that under normal growth conditions many E2F7-expressing cells displayed decreased nucleolar UBF levels and altered localisation (Fig. 6a; compare E2F7 transfected vs non-transfected cell, marked with arrows bottom panel). This suggested that E2F7 causes nucleolar segregation as a result of its ability to inhibit rRNA gene transcription^8^. To explore this idea further, we examined nucleophosmin (NPM) localisation in the presence of E2F7, as NPM has been shown to relocalise from the nucleolus to the nucleoplasm during stress^27^. Indeed, ectopic E2F7 resulted in nucleolar segregation as visualised by dispersal and relocalisation of NPM (Fig. 6b). Notably, E2F7 also resulted in the relocalisation of the nucleolar protein nucleolin in the absence of stress (Fig. 6c). This occurred to a similar extent to that observed when Pol I was inhibited with actinomycin D (SI Fig. 3A) and was dependent on an intact DNA-binding domain (Fig. 6c). Similar results were also seen with GFP-tagged E2F7, but not GFP alone (SI Fig. 3B). Thus, E2F7 can impair nucleolar structure and inhibit rRNA transcription.

## Discussion

Cancer cells are by nature rapidly dividing and as such require elevated protein production mediated by enhanced rRNA gene transcription and Pol I activity^28^. The regulation of Pol I activity is a key determinant of ribosome biogenesis as it provides rRNA for the mature ribosome^1,2^. Moreover, rRNA gene transcription is commonly deregulated in cancer through oncogenic events^28^. As Pol I activity is high in cancer and it has been shown that maintaining elevated Pol I activity is critical for cancer cell survival, it is an attractive therapeutic target^29^. Thus, uncovering new pathways responsible for regulating Pol I activity could provide novel therapeutic options to treat human cancer. In this study we demonstrate that the atypical E2F family member, E2F7, is able to repress Pol I transcriptional activity and impair nucleolar structure resulting in a decrease in rRNA transcription and global protein production.

We have found, surprisingly, that E2F7 is able to inhibit Pol I activity through modulating UBF recruitment to the Pol I promoter (Fig. 6d). UBF is necessary for Pol I activation and key for upregulation of rRNA gene transcription^21,22^. E2F activity has been implicated in the regulation of Pol I, where E2F1 binds to the rRNA gene promoter to modulate its activity^30^. More recently, the E2F target gene CDC6 product was shown to regulate rRNA gene transcription initiation, suggesting a mechanism by which DNA replication and transcription are coordinated^31^. The fact that E2F7, a negative regulator of E2F1 activity and an important regulator of DNA replication^14–17^, results in the negative regulation of Pol I support this. Moreover, in certain tumour cell lines, particularly those derived from haematopoietic and lymphoid malignancies, E2F7 is expressed at very low levels (www.cbioportal.org/index.do). Interestingly, in clinical disease, such as glioblastoma, E2F7 is similarly expressed at lower levels relative to normal tissue (https://www.oncomine.com). The reduced level of E2F7 may therefore contribute to enhanced ribosome biogenesis and protein synthesis characteristic of malignant disease.

During the cell cycle, the nucleolus undergoes extensive changes at the onset of mitosis and rDNA transcription is inhibited between prometaphase and telophase^32^. While our data suggest that the effects of E2F7 on Pol I activity and thus protein synthesis can be uncoupled from cell cycle regulation, the ability of E2F7 to co-ordinate E2F activity with growth signals may be an additional important mechanism, which allows E2F7 to exert additional effects on cell growth. For example, during DNA damage E2F7 is upregulated where it is involved in DNA repair^18,19^, which could provide a means for coordinating cell cycle arrest, promoting DNA r1epair and preventing protein synthesis.

Together, our study suggests that enhanced levels of E2F7 lead to inhibition of Pol I activity and nucleolar segregation. This provides an important mechanism during the cellular response to stress to co-ordinate protein synthesis and cell cycle arrest. Deregulated E2F7 activity in cancer would thus be expected to impact on tumour cell growth both through its ability to modulate E2F1 activity as well as by influencing protein production through altered Pol I activity.

## Materials and Methods

### Plasmids, antibodies and reagents

The following plasmids have been previously described: pcDNA-HA-E2F7, pcDNA-HA-E2F7 R185A, R334A, R185A/R334A, CMV-E2F1 and E2F1-luciferase^16,18,19^. Mouse anti-HA antibody HA11 was from BAbCO. Anti-puromycin antibody was from Merck Millipore. Goat anti-E2F7, rabbit anti-E2F7 H300, rabbit anti-UBF, mouse anti-nucleophosmin and mouse anti-nucleolin antibodies were from Santa Cruz. Rabbit anti-E2F7 antibody was from Proteintech. Mouse anti-actin, anti-BrdU BU-33 and anti-Flag antibodies were from Sigma. HRP-conjugated secondary antibody was from DAKO. Alexa Fluor-conjugated secondary antibodies were from Molecular Probes. Actinomycin D was from Sigma. EBSS growth medium was from ThermoFisher Scientific.

### Cell lines and transfections

U2OS and MCF7 cells were grown in 5% FCS-DMEM plus antibiotics under 5% CO_2_ unless otherwise denoted. Plasmid transfections were performed using GeneJuice (Merck Biosciences). siRNA transfections were performed using Oligofectamine (Invitrogen) using 25 nM siRNA. Human E2F7 siRNA has been previously described^18^ and siRNA targeting GFP was used as a non-targeting control.

### Immunostaining

Cells were seeded onto 13 mm glass coverslips and fixed with 3.7% formaldehyde. Permeabilisation was performed for 5 min with 0.5% Triton X-100 in PBS followed by incubation with primary antibody for an hour at room temperature (or overnight). Coverslips were washed with 0.025% Tween in PBS extensively before adding secondary antibody. Coverslips were mounted on microscope slides using Vectashield with or without DAPI (4,6-diamino-2-phenylindole). Images were obtained using an Olympus BX51 inverted fluorescence microscope using a ×63 oil-immersion lense. To visualise nascent RNA, U2OS cells grown on coverslips were labelled with 2 mM fluorouridine (FUrd) for 30 min before fixing with 1% formaldehyde and staining with BrdU antibody BU-33 (Sigma).

### FACS analysis

Cells were seeded into 6 cm dishes and treated as appropriate before harvesting. Growth media were collected and adherent cells were lifted by adding 1 mL of trypsin per dish. The cells were pelleted (800 × *g*) for 5 min at 4 °C and washed once with PBS. The cells were fixed in ice-cold 70% ethanol/PBS (v/v). Fixed cells were washed with PBS and stained in 2% (v/v) propidium iodide in the presence of 125 U/mL DNAse-free RNAse A. Stained cells were analysed using flow cytometry (Accuri C6, BD Bioscience).

### Puromycin incorporation assays

Cells were treated with either vehicle or cyclohexamide (100 μg/mL) for 10 min before washing out and replacing with fresh medium containing 10 μg/mL puromycin. Puromycin incorporation was carried out for 2–5 min at 37 °C before harvesting.

### Chromatin immunoprecipitation

Cells were fixed with 1% formaldehyde for 10 min, quenched with 0.125 M glycine followed by permeabilisation in SDS lysis buffer (1% SDS, 10 mM EDTA, 50 mM Tris, pH 8.0.). Samples were sonicated using Diagenode Bioruptor for 10–20 cycles of 30 s on 30 s off. Immunoprecipitated complexes were collected using 25 μL protein A/G slurry. Samples were washed extensively with low salt buffer (0.1% SDS, 1% Triton, 2 mM EDTA, 20 mM Tris, pH 8, 150 mM NaCl) and LiCl wash buffer (10 mM Tris pH 8.0, 1% Na-deoxycholate, 1% NP-40, 250 mM LiCl, 1 mM EDTA). Complexes were washed a further two times with TE before reverse cross-linking and RNAse digestion at 65 °C for 3 h followed by overnight at 55 °C. DNA was isoloated using Qiagen columns according to the manufacturer’s instructions. ChIP samples along with inputs were analysed using either standard RT-PCR protocols or taken on to qPCR using SYBR green II. The following primer sets were used: hrDNA: A forward: CCGTGGGTTGTCTTCTGACT reverse: AAGCGAAACCGTGAGTCG B forward CAGCGCGCCGTAGCTC reverse. GCCTCAGACGGCCAGGAT C forward: GATCCTTTCTGGCGAGTCC reverse: GGAGCCGGAAGCATTTTC. D forward: GTGTGTGGCTGCGATGGT reverse: CCAACCTCTCCGACGACAG. E forward: CGACCTGTCGTCGGAGAG reverse: GGACGCGCGAGAGAACAG. Actin forward: ATCGTGCGTGACATTAAGGAGAAG reverse: CTGGAAGCAGCCGTGGCCGTCTCTTG. E2F1 forward: AGGGCTCGATCCCGCTCCG reverse: TAAAGCCAATAGGAACCGCCG Cdc6 forward: GGCCTCACAGCGACTCTAAGA reverse: CTCGGACTCACCACAAGC.

### Reverse transcription qPCR

RNA was isolated using ReliaPrep kit (Promega) according to the manufacturer’s instructions before cDNA synthesis using random hexamers. qPCR was performed using SYBR Green and quantified using the 2^−ΔΔCT^ method. Pre-rRNA signals were normalised to GAPDH. Primers targeting the pre-rRNA were set A: forward: GAACGGTGGTGTGTCGTT reverse: GCGTCTCGTCTCGTCTCACT. Set B: forward: GCCTTCTCTAGCGATCTGAGAG reverse: CCATAACGGAGGCAGAGACA.

## Electronic supplementary material

SI Figure 1

SI Figure 2

SI Figure 3

SI Figure legends
