# Supplementary figures and images for: Functional interplay between E2F7 and ribosomal rRNA gene transcription regulates protein synthesis
Source: Cell Death Dis. 2018 May 14;9(5):577. doi: 10.1038/s41419-018-0529-6 (PMC5951837; doi:10.1038/s41419-018-0529-6)

SI Figure 1

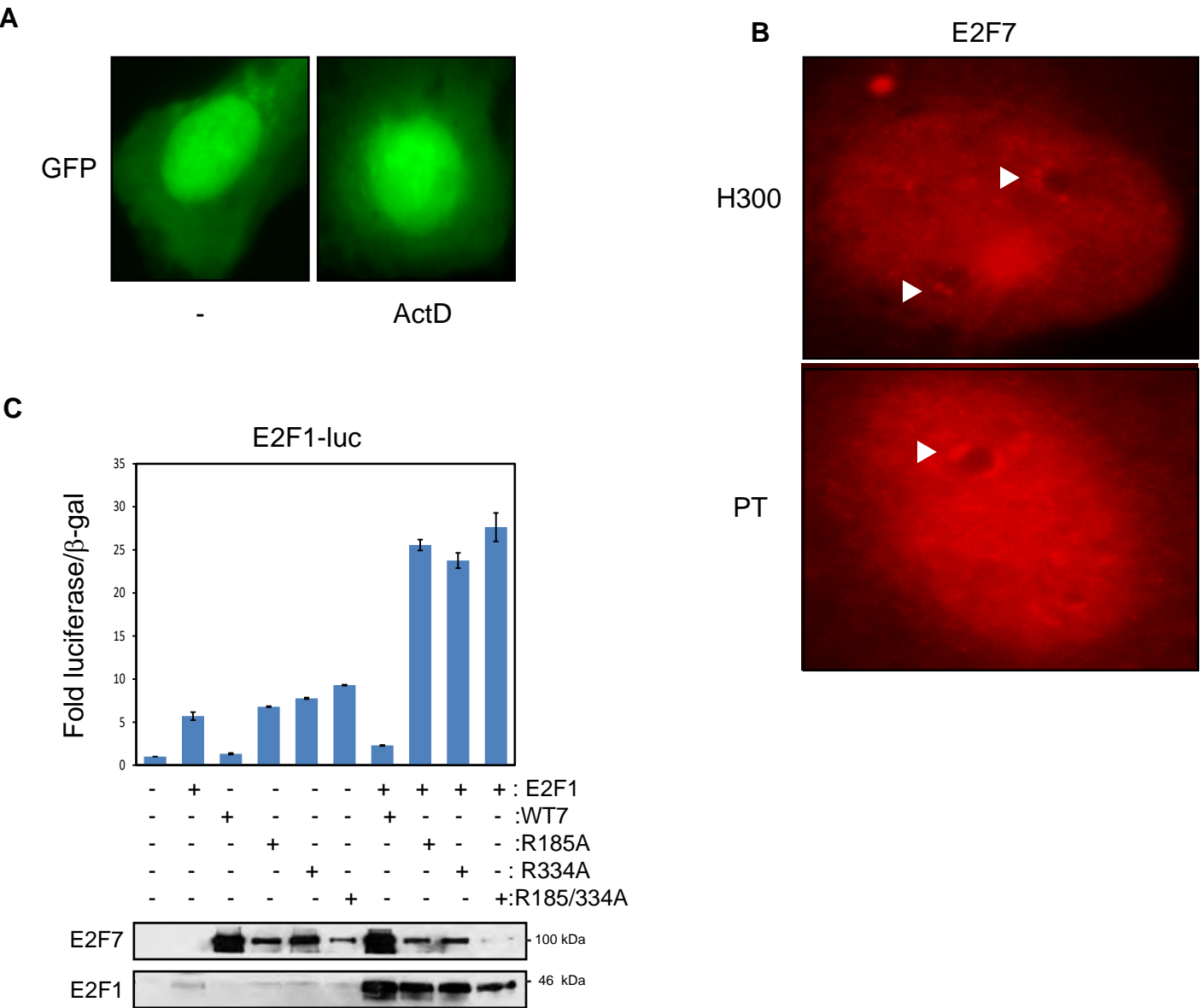

Supplement: Supplementary file 1 — SI Figure 1 [file 41419_2018_529_MOESM1_ESM.pdf]

Supplementary Figure 2

A

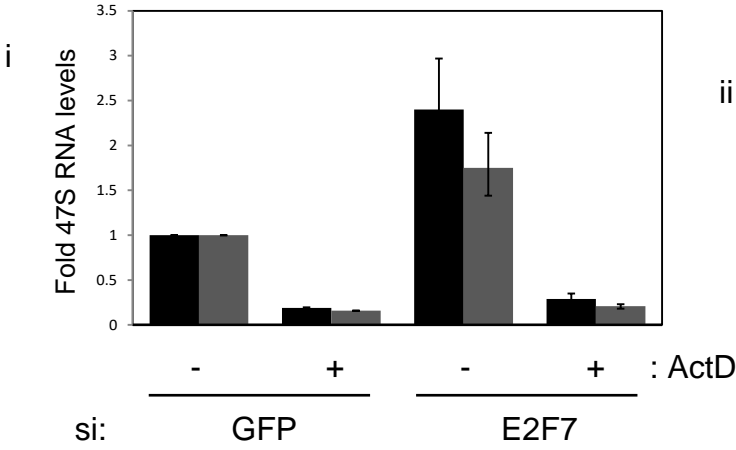

ii

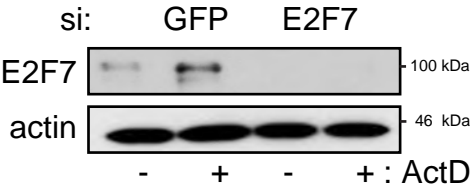

B

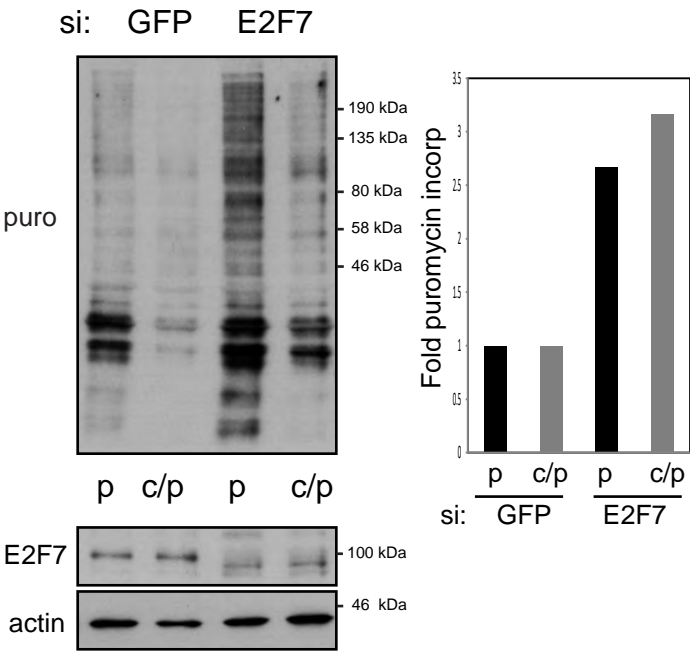

C

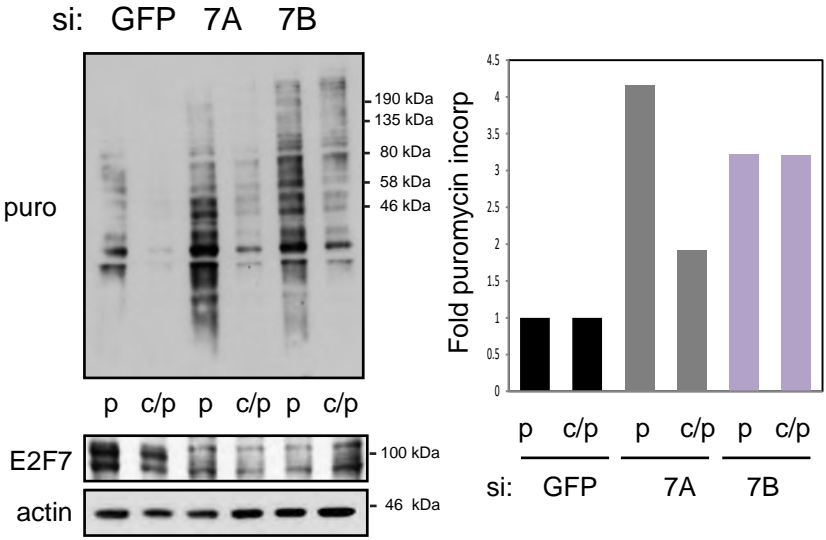

Supplement: Supplementary file 2 — SI Figure 2 [file 41419_2018_529_MOESM2_ESM.pdf]

Supplementary Figure 3

A

ActD: nucleophosmin

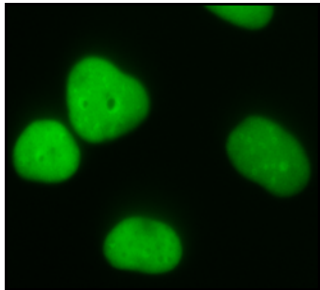

B

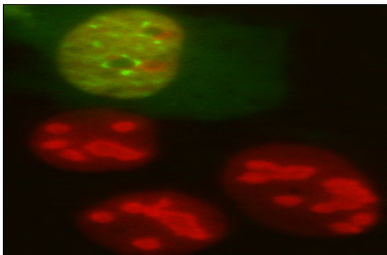

GFP-E2F7  
nucleophosmin

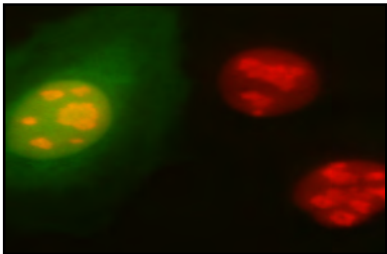

GFP-vector  
nucleophosmin

Supplement: Supplementary file 3 — SI Figure 3 [file 41419_2018_529_MOESM3_ESM.pdf]
