# Supplementary material for: Functional interplay between E2F7 and ribosomal rRNA gene transcription regulates protein synthesis
Source: Cell Death Dis. 2018 May 14;9(5):577. doi: 10.1038/s41419-018-0529-6 (PMC5951837; doi:10.1038/s41419-018-0529-6)
Supplement: Supplementary file 4 — SI Figure legends [file 41419_2018_529_MOESM4_ESM.docx]

**Supplementary Figure Legends.**

**Supplementary Figure 1.**

**A.** U2OS cells expressing p-EGFP-C1 were treated with (Act D) or without (-) actinomycin D (1 nM) overnight. **B.** U2OS cells were treated with 1 nM actinomycin D overnight before fixation and processing for immunofluorescence. Endogenous E2F7 was detected using anti-E2F7 antibody H300 (Santa Cruz) or anti-E2F7 antibody PT (Proteintech). Arrowheads denote E2F7 localisation at nucleolar cap region. **C.** E2F-1 luciferase assays were performed in U2OS cells transfected with the appropriate HA-E2F7 constructs as denoted, with or without E2F-1. Cells were co-transfected with β-galactosidase which was used to normalise luciferase values. WT7 = wild-type E2F7.

**Supplementary Figure 2.**

**A.** U2OS cells were treated with control (GFP) or E2F7 siRNA for 72h along with vehicle control (-) or 1 nM actinomycin D overnight (Act D), before RNA isolation and RT-qPCR. 47S transcript levels were quantified using two different primer sets (Black bars = A and grey bars = B) and results are expressed as fold over control after normalising for GAPDH levels. Graph represents mean +/- s.e.m. **B.** MCF7 cells were treated with control (GFP) or E2F7 siRNA for 72h before performing puromycin incorporation assays. Graph represents the level of puromycin incorporation after normalising for actin. p, puromycin; c/p, cyclohexamide/puromycin. **C.** MCF7 cells were treated with control (GFP) or E2F7 (A and B) siRNA for 72h before performing puromycin incorporation assays. P, puromycin; c/p, cyclohexamide/puromycin. Graph represents fold puromycin incorporation after normalising to actin.

**Supplementary Figure 3. A.** U2OS cells were treated with 1 nM actinomycin D (Act D) overnight before fixation and processing for immunofluorescence. **B.** U2OS cells were transfected with either GFP-wild-type E2F7 (GFP-E2F7) or GFP-empty vector (GFP-vector).
